# Testing Theory-Enhanced Messaging to Promote COVID-19 Vaccination Among Adults: Randomized Controlled Trial

**DOI:** 10.2196/79228

**Published:** 2025-10-07

**Authors:** Rachael Piltch-Loeb, Yanhan Shen, Sasha Fleary, McKaylee Robertson, Josefina Nuñez Sahr, Kate Penrose, Jenna Sanborn, Surabhi Yadav, Avantika Srivastava, Denis Nash, Angela Parcesepe

**Affiliations:** 1 Institute for Implementation Science in Population Health City University of New York New York, NY United States; 2 Department of Environmental, Occupational, Geospatial Health Sciences City University of New York New York City, NY United States; 3 Department of Community Health and Social Sciences Graduate School of Public Health and Health Policy City University of New York New York City United States; 4 Department of Epidemiology and Biostatistics Graduate School of Public Health and Health Policy City University of New York New York, NY United States; 5 Maternal and Child Health Gillings School of Global Public Health University of North Carolina at Chapel Hill Chapel Hill United States

**Keywords:** COVID-19, vaccine hesitancy, behavioral interventions, public health communication, video interventions, inoculation theory

## Abstract

**Background:**

Uptake of the COVID-19 vaccine has been low in the United States despite ongoing public health recommendations. This has been linked to many factors, including pandemic fatigue; reduced risk perception; dis- and misinformation; and, more recently, symptoms of depression and anxiety. Novel communication and messaging strategies are one potential approach to promote vaccine uptake.

**Objective:**

This randomized controlled trial aimed to fill research gaps by testing the effect of 2 communication-based approaches<strong>―</strong>the use of a short attitudinal inoculation message and cognitive behavioral therapy (CBT) kernel messaging<strong>―</strong>compared to standard public health messaging on vaccine uptake in a cohort of adult US residents.

**Methods:**

We completed a 3-arm, parallel-group, assessor-blinded stratified randomized trial between April 15, 2024, and May 2, 2024. Individuals were eligible if they were aged ≥18 years and (1) had received at least one dose of the COVID-19 vaccine but (2) had not received COVID-19 vaccine doses since September 11, 2023, and (3) had not been infected with SARS-CoV-2 in the previous 3 months. We purposively sampled eligible individuals with and without symptoms of anxiety and depression. Participants were randomly allocated to the (1) attitudinal inoculation intervention, (2) CBT kernel intervention, or (3) standard public health messaging intervention.

**Results:**

By the 4-week follow-up, COVID-19 vaccination uptake was low overall (17/1403, 1.2%, 95% CI 0.6%-1.8%) and did not significantly differ by study arm<strong>―</strong>1.5% (7/469) in the CBT kernel arm (95% CI 0.4%-2.8%), 0.9% (4/466) in the inoculation arm (95% CI 0%-1.8%), and 1.3% (6/468) in the standard arm (95% CI 0.3%-2.4%). Compared to the standard arm, the CBT kernel intervention yielded a risk difference (RD) of 0.3% (95% CI −1.3% to 1.8%) and a risk ratio (RR) of 1.21 (95% CI 0.41-3.59); the inoculation intervention yielded an RD of −0.4% (95% CI −1.8% to 1%) and an RR of 0.69 (95% CI 0.19-2.44). Reported SARS-CoV-2 infections and vaccine uptake did not differ by anxiety or depression symptoms. At baseline, approximately one-third of participants (466/1403, 33.21%) reported high willingness to receive another COVID-19 vaccine dose, with no significant differences across arms. At the 4-week follow-up, willingness remained similar across groups (CBT kernel vs standard arm: RD=−0.3%, 95% CI −6.3% to 5.8%, and RR=0.99, 95% CI 0.79-1.25; inoculation vs standard arm: RD=7%, 95% CI 0.8%-13.3%, and RR=1.23, 95% CI 0.98-1.53). Willingness did not differ by mental health status.

**Conclusions:**

Successful efforts to increase uptake of the COVID-19 vaccine via theory-enhanced messaging remain elusive. Findings underscore the challenges of shifting behavior through messaging alone in a context of declining public trust and a diminished sense of urgency years after the onset of the COVID-19 pandemic. Ongoing research is needed to better understand and address informational and behavioral barriers to vaccination.

**Trial Registration:**

ClinicalTrials.gov NCT06119854; https://clinicaltrials.gov/study/NCT06119854

## Introduction

### Background

Within the United States, uptake of the updated COVID-19 vaccine remains suboptimal**―**as of June 8, 2024, only 22% of adults and 15% of children aged <17 years had received the latest vaccine for the 2023 to 2024 season [1]. People who did not intend to receive a COVID-19 vaccine increased from 37% in September 2023 to 43% in June 2024 despite ongoing COVID-19 transmission, illness, and mortality [2,3].

Effective public health communication is essential to improve vaccination rates, particularly messages and interventions that can lead to changes in behavior, not just attitudes. There is limited evidence on the effectiveness of communication-based interventions designed to address vaccine hesitancy, and such studies rarely include vaccine uptake as an outcome [4,5]. In addition, the COVID-19 pandemic has triggered a sharp rise in anxiety and depression symptoms [6], whereas mental health symptoms increase the risk of severe COVID-19 outcomes and are linked to a lower likelihood of receiving a COVID-19 vaccine [7,8]. Little is known about the effectiveness of vaccine uptake interventions among this population.

Novel messaging strategies may be warranted to promote vaccine uptake and reduce vaccine hesitancy. *Attitudinal inoculation* offers a promising approach as a brief, scalable strategy that uses the power of narrative, values, and emotion to bolster resistance to misinformation and reduce vaccine hesitancy [9,10]. A quasi-experimental trial tested the efficacy of attitudinal inoculation videos to enhance COVID-19 vaccine acceptance**―***inoculated* participants showed an improved ability to recognize rhetorical strategies used in misinformation, were less likely to subsequently share false information, and exhibited greater vaccination willingness [11]. Although this approach has been shown to decrease COVID-19 *vaccine* *hesitancy*, the extent to which it increases *vaccination* remains unknown.

A more tailored approach may be warranted for adults with symptoms of depression and anxiety. Cognitive behavioral therapy (CBT), which focuses on building problem-solving skills and addressing distorted thoughts, overwhelming emotions, and maladaptive behaviors, has been shown to reduce psychological distress [12]. Applying this to vaccine misinformation and hesitancy in this population may serve as a behavioral vaccine, encouraging the adoption of COVID-19 prevention measures. While Embry [13] proposed the use of “evidence-based kernels” to improve mental health through behavioral vaccines and others have suggested integrating public health messaging with psychological treatments such as CBT during the pandemic [12], to our knowledge, this approach has not been formally assessed.

### Objectives

This study aimed to fill research gaps by testing the effect of attitudinal inoculation and CBT kernel messaging approaches on vaccine uptake among US residents. The specific aims were to compare the effect of (1) attitudinal inoculation versus standard messaging and (2) CBT kernel messaging versus standard messaging on COVID-19 vaccination and vaccination willingness and compare the effect of each messaging strategy on vaccine uptake among individuals with and without moderate or severe symptoms of anxiety or depression.

## Methods

### Trial Design

We conducted a 3-arm, parallel-group, assessor-blinded, stratified randomized trial. Recruitment and randomization started on April 15, 2024, and continued until May 2, 2024. Outcome assessment at the 4-week follow-up started on May 13, 2024, and was completed by June 13, 2024. After consenting, enrolled participants were randomly assigned to one of three arms**―**(1) attitudinal inoculation, (2) CBT kernels, or (3) standard public health messaging**―**at a ratio of 1:1:1. Each arm was also stratified by presence or absence of moderate to severe anxiety or depression symptoms, defined by scoring ≥10 on the Patient Health Questionnaire–8 [14] or 7-item Generalized Anxiety Disorder scale [15] between December 2022 and December 2023. Intervention delivery and data collection took place fully web based.

### Recruitment

Individuals were recruited from the Communities, Households, and SARS-CoV-2 Epidemiology (CHASING) COVID Cohort Study, a diverse community-based sample of adults aged ≥18 years who reside in the United States or US territories and who enrolled in the cohort between March 28, 2020, and August 21, 2020. Details of recruitment into the CHASING COVID Cohort Study and follow-up have been described elsewhere [16]. Briefly, participants in the cohort completed approximately quarterly online assessments related to health and behaviors, SARS-CoV-2 infection history, and COVID-19 vaccination status since March 2020. From this cohort, a sample of 2411 participants who met the eligibility criteria were contacted via email and SMS text message with a recruitment message that included a link to the initial survey. This message prompted individuals to complete a brief screener to confirm their eligibility before proceeding to the full survey.

### Participants

Participant eligibility criteria at the time of study enrollment (April 2024-June 2024) were (1) age of ≥18 years; (2) ability to read in English; (3) current residence in the United States; (4) completion of at least one survey as part of the CHASING COVID Cohort Study between December 7, 2022, and December 22, 2023; and (5) undervaccination, defined as having received at least one dose of the COVID-19 vaccine but none since September 11, 2023. Informed by our previous research [17], we focused on individuals with at least one COVID-19 vaccine dose—the *moveable middle*—who showed some willingness to get vaccinated and may be more responsive to a brief video intervention than those unvaccinated. Participants were excluded if they (1) had had a SARS-CoV-2 infection in the previous 3 months; (2) had never received *any* dose of a COVID-19 vaccine; or (3) were flagged for fraudulent behavior, such as suspicious response times. There was no racial or gender bias in the selection of participants.

### Ethical Considerations

This study received approval from the institutional review board of the City University of New York Graduate School of Public Health and Health Policy (protocol NCT06119854) and followed the CONSORT (Consolidated Standards of Reporting Trials) social and psychological intervention reporting guidelines [18,19]. Informed consent forms were completed via web browser. Participants were compensated with a total of US $50 in gift cards for completing both surveys. All study data were stored on encrypted drives, and any identifiable information (eg, names) was stored separately from study data. Only members of the study team had access to survey data.

### Intervention and Control Conditions

Participants viewed 1 of 3 videos, all limited to <1 minute so as to keep participants’ attention and be consistent with standard commercials that participants may see on television [20]. The video content of the experimental arms was developed using formative mixed methods research, including a pretrial survey on vaccine perceptions and qualitative interviews with participants reporting anxiety or depression. This revealed a common concern about vaccine effectiveness as a broader “meta-narrative” [17]. Hence, the inoculation video aimed to build resistance to misinformation about vaccine effectiveness by refuting the information using facts and logic, a strategy designed to pre-emptively strengthen counterarguments. Given that participants with anxiety or depressive symptoms were more likely to cite not *making time* as a reason for being behind on vaccination, the CBT kernel condition focused on connecting maladaptive thoughts and reasoning with behaviors, modeling stated versus revealed preferences, and reframing maladaptive reasoning to influence behaviors. Script development was guided by the evidence-based kernels by Embry [13] and informed by consultation with a psychologist and psychiatrist.

Specifically, the inoculation video addressed concerns about vaccine effectiveness, focused on bolstering resistance to mis- and disinformation. The CBT kernel video focused on addressing cognitive barriers to vaccination, specifically maladaptive reasoning to get vaccinated. The standard public health messaging arm was a brief video adapted from existing public health service announcements, with no inoculation or CBT kernel elements. The videos were professionally created by Long Story Short, a production company with experience in public service announcement creation. Further details, including a description and scripts of these videos, can be found in Multimedia Appendix 1.

After receiving the brief digital intervention, participants received 2 reminders to get vaccinated via SMS text message or email on the first and third days after completing the baseline assessment. Messages were tailored to each arm and included a link to a COVID-19 vaccination venue locator (Multimedia Appendix 1).

### Stratified Randomization Procedure and Masking

#### Randomization Procedure

We used a 2-stage procedure for randomization using the Qualtrics built-in *Randomizer* tool (Qualtrics International Inc). First, participants were stratified into 2 groups based on the presence or absence of symptoms of anxiety or depression. Second, participants were randomized to 1 of 3 intervention arms upon being enrolled.

#### Masking

Two team members involved in study operations and study implementation (AS and JNS) were not blinded to study arm assignments. All other team members, including investigators and the data analyst (JS), were blinded to study arm assignments.

### Measures

#### Intervention Adherence Measure

The survey was programmed to require participants to remain on the video page for the duration of the video. However, time spent on the web page does not necessarily reflect attentiveness; hence, participants were also asked the following**―**“Did this video hold your attention?”**―**with the response options “Yes, it held my attention” and “No, it did not hold my attention/I did not watch the video.”

#### Primary Outcome: Receipt of COVID-19 Vaccine Dose at 4 Weeks

In the 4-week survey, participants were asked the following: “Have you received a COVID-19 vaccine dose since [date of intervention]?” Participants who responded *yes* were considered vaccinated.

#### Secondary Outcome: Vaccination Willingness at 4 Weeks

In the 4-week survey, participants who had not received a vaccine since the intervention were asked the following**―**“How willing are you to receive another COVID-19 vaccine dose?”**―**with the following response options: “Very willing,” “Somewhat willing,” “Not willing,” or “Don’t know.” Participants who responded “Very willing” were defined as vaccination willing.

#### Pretrial Vaccination Willingness

After enrollment but before randomization, participants were asked the following**―**“How willing are you to receive another COVID-19 vaccine dose?”**―**with the following response options: “Very willing,” “Somewhat willing,” “Not willing,” or “Don’t know.”

#### Posttrial Vaccination Willingness

Immediately after the intervention, participants were asked the following**―**“How likely are you to make time to get a vaccine in the next month?”**―**with the following response options: “Very likely,” “Somewhat likely,” “Not likely,” and “Don’t know/not sure.” They were also asked the following**―**“Are you planning to make an appointment to get the COVID-19 vaccine in the next month?”**―**with the following response options: “Already made an appointment,” “Planning,” “Not planning,” “Don’t know/not sure.” These questions were asked again in the 4-week follow-up survey to participants who had not received an additional vaccine dose.

#### Post Hoc Stratification Variables

Post hoc stratification analyses were conducted to assess differences in primary and secondary outcomes based on susceptibility to severe COVID-19, worry about COVID-19, and pretrial perceptions of vaccine efficacy. Details regarding the measurement of stratification variables can be found in Multimedia Appendix 2.

### Statistical Analysis

We present summaries of characteristics using standardized mean differences (SMDs) by treatment assignment. Primary analyses were conducted under the intention-to-treat (ITT) principle including all participants who underwent randomization. Strict ITT analysis is hard to achieve for 2 reasons: missing outcomes for participants and protocol nonadherence. For the primary analysis, we used multiple imputation for those lost to follow-up at some point after randomization (as recommended by the CONSORT guidelines [20]).

For the primary outcome, we generated risk ratios (RRs) using a robust Poisson regression model and generated risk differences (RDs). For the secondary outcome, we generated RRs using a Poisson regression model. The model for the overall effect estimation included the randomization arms and a term for the presence or absence of symptoms of anxiety or depression [21,22].

We assessed the effect of the intervention among those with or without symptoms of anxiety or depression. The stratified models included the randomization arms as the independent variable. Post hoc stratification analyses for the primary and secondary outcomes were conducted separately among subsets of participants based on the presence or absence of the post hoc stratification factors (susceptibility to severe COVID-19, worry about COVID-19, and pretrial perceptions of vaccine efficacy). The model included the randomization arms and presence or absence of symptoms of anxiety or depression.

The validity of an ITT effect estimate requires correct adjustment for selection bias due to differential loss to follow-up and missing outcome data [21,23,24]. The methods for sensitivity analysis can be found in Multimedia Appendix 3. We used multiple imputation for loss to follow-up and missing data, and details can be found in Multimedia Appendix 4. Sample size calculation can also be found in Multimedia Appendix 4.

## Results

### Study Sample

**Figure 1:**
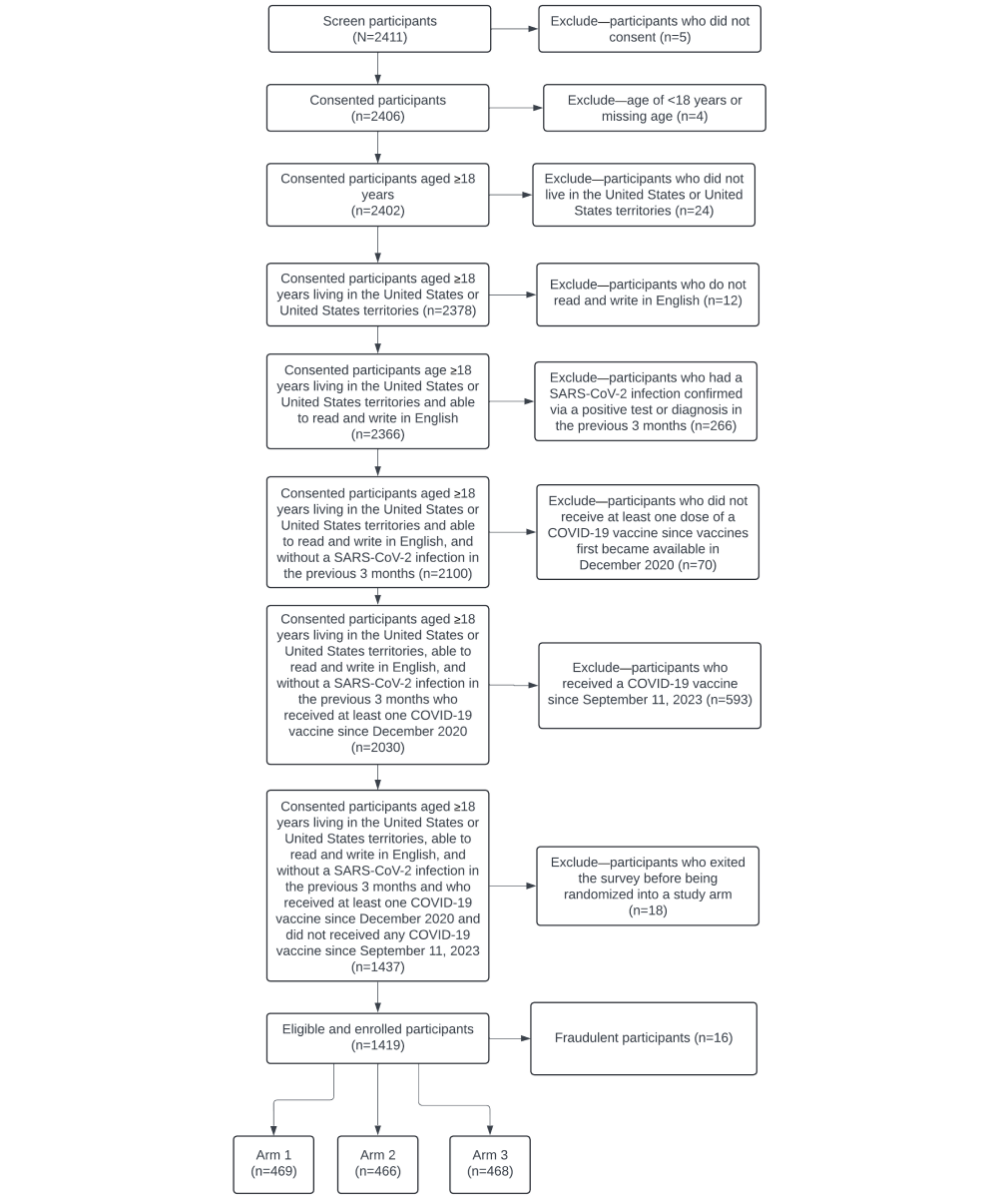
CONSORT Flow Diagram.

Overall, 2411 CHASING COVID Cohort Study participants were invited, and 1419 (58.86%) were eligible and randomized to the intervention. After completing the 4-week survey, of the 1419 participants, 16 (1.13%) were removed from the sample based on data quality protocols, leaving an analytic sample of 1403 (98.87%) participants. Randomization resulted in 33.43% (469/1403) of the individuals in the CBT kernel arm, 33.21% (466/1403) in the inoculation arm, and 33.36% (468/1403) in the standard public health messaging arm (Figure 1).

Briefly, most participants were female (787/1403, 56.09%) and non-Hispanic White individuals (842/1403, 60.01%), had at least a college education (811/1403, 57.8%), were employed (1011/1403, 72.06%), and had employer-based health insurance (783/1403, 55.81%). Most (924/1403, 65.86%) did not report having any comorbidities. The median age of the participants was 41 (IQR 33-54) years, with 17.03% (239/1403) aged ≥60 years. As of December 2023, participants reported a median of 3 COVID-19 vaccine doses, 74.34% (1043/1403) reported having a primary care provider, and 38% (533/1403) had moderate to severe symptoms of anxiety or depression. Participants were balanced between groups on nearly all measured characteristics (SMD<0.2) except for reported discrimination in a health care experience (SMD 0.235). Before the intervention, most (982/1403, 70%) participants were willing or very willing to receive another COVID-19 vaccine dose, with differences in vaccination willingness across arms. Loss to follow-up was minimal (56/1403, 4%) and did not differ by arm (*P*>.90).

### Postintervention Perceptions

Immediately following the intervention, participants were asked whether the video held their attention, with no significant differences across arms (*P*=.60). In total, 9.69% (136/1403) of the participants reported that they were very likely, 20.74% (291/1403) reported that they were somewhat likely, and 57.38% (805/1403) were unlikely to make time to get vaccinated, with no significant differences across arms (*P*=.86; Table 1). Immediately after the intervention, 15.89% (223/1403) of the participants indicated that they were planning to make a vaccine appointment, with no observed difference between arms (*P*=.85; Table 1). At the 4-week follow-up, the same patterns held for willingness to make time to get vaccinated and likelihood to make a vaccine appointment. In addition, most participants (1214/1403, 86.53%) reported no barriers to making a vaccine appointment, with no differences between arms (*P*=.40; Table 1).

**Table 1:** Measures of likelihood of making time to get vaccinated and making an appointment for a vaccine (N=1403)^a^.

		Overall, n (%)	CBT^b^ arm (n=469), n (%)	Inoculation arm (n=466), n (%)	Standard arm (n=468), n (%)	*P* value	
**Vaccination willingness (before the intervention)**							―^c^
	Did not know or not sure	102 (7.3)	32 (6.8)	38 (8.2)	32 (6.8)		
	Not willing	319 (22.7)	108 (23)	88 (18.9)	123 (26.3)		
	Somewhat willing	537 (38.3)	188 (40.1)	171 (36.7)	178 (38)		
	Very willing	445 (31.7)	141 (30.1)	169 (36.3)	135 (28.8)		
**Making time for vaccination**―**after the intervention**							.86
	Did not know or not sure	167 (11.9)	61 (13)	53 (11.4)	53 (11.3)		
	Not likely	805 (57.4)	274 (58.4)	258 (55.4)	273 (58.6)		
	Somewhat likely	291 (20.7)	87 (18.6)	107 (23)	97 (20.7)		
	Very likely	136 (9.7)	46 (9.8)	46 (9.9)	44 (9.4)		
	Missing data	4 (0.3)	1 (0.2)	2 (0.4)	1 (0.2)		
**Making time for vaccination**―**4-week follow-up**							.37
	Did not know or not sure	128 (9.1)	44 (9.4)	47 (10.1)	37 (7.9)		
	Not likely	847 (60.4)	295 (62.9)	261 (56)	291 (62.2)		
	Received vaccine	17 (1.2)	7 (1.5)	4 (0.9)	6 (1.3)		
	Somewhat likely	304 (21.7)	91 (19.4)	110 (23.6)	103 (22)		
	Very likely	106 (7.6)	32 (6.8)	43 (9.2)	31 (6.6)		
	Missing data	1 (0.1)	0 (0)	1 (0.2)	0 (0)		
**Making an appointment for a vaccine**―**after the intervention**							.85
	Already made an appointment	24 (1.7)	9 (1.9)	8 (1.7)	7 (1.5)		
	Did not know or not sure	417 (29.7)	146 (31.1)	139 (29.8)	132 (28.2)		
	Not planning	737 (52.5)	244 (52)	236 (50.6)	257 (54.9)		
	Planning	223 (15.9)	69 (14.7)	82 (17.6)	72 (15.4)		
	Missing data	2 (0.1)	1 (0.2)	1 (0.2)	0 (0)		
**Making an appointment for a vaccine**―**4-week follow-up**							.40
	Already made an appointment	18 (1.3)	9 (1.9)	6 (1.3)	3 (0.6)		
	Did not know or not sure	329 (23.4)	116 (24.7)	114 (24.5)	99 (21.2)		
	Not planning	814 (58)	272 (58)	259 (55.6)	283 (60.5)		
	Planning	224 (16)	65 (13.9)	82 (17.6)	77 (16.5)		
	Received vaccine	17 (1.2)	7 (1.5)	4 (0.9)	6 (1.3)		
	Missing data	1 (0.1)	0 (0)	1 (0.2)	0 (0)		
**Number of vaccination barriers**							.40
	No barriers	1214 (86.5)	411 (87.6)	393 (84.3)	410 (87.6)		
	1 barrier	164 (11.7)	49 (10.4)	66 (14.2)	49 (10.5)		
	2 barriers	24 (1.7)	9 (1.9)	7 (1.5)	8 (1.7)		
	3 barriers	1 (0.1)	0 (0)	0 (0)	1 (0.2)		

^a^Standardized mean difference for vaccination willingness (before the intervention): 0.159 and 0.077. The two values represent the two separate comparisons: CBT arm versus standard arm and inoculation arm versus standard arm, respectively.

^b^CBT: cognitive behavioral therapy.

^c^Not applicable.

### Primary Outcome: Vaccination at 4 Weeks

The total number of vaccinations by the 4-week follow-up was low (17/1403, 1.21%, 95% CI 0.6%-1.8%) and did not significantly differ by arm**―**1.5% (7/469) in the CBT kernel arm (95% CI 0.4%-2.8%), 0.9% (4/466) in the inoculation arm (95% CI 0%-1.8%), and 1.3% (6/468) in the standard arm (95% CI 0.3%-2.4%; Table 2). There were no differences in vaccination among those with and without anxiety or depression symptoms. Reported SARS-CoV-2 infections were balanced across arms. Compared to the standard condition, the CBT kernel intervention yielded an RD of 0.3% (95% CI −1.3% to 1.8%) and an RR of 1.207 (95% CI 0.406-3.59). The inoculation intervention yielded an RD of −0.4% (95% CI −1.8% to 1%) and an RR of 0.687 (95% CI 0.194-2.436) relative to the standard condition.

**Table 2:** Primary outcome―vaccine uptake at the 4-week follow-up by intervention arm and presence of mental health symptoms.

Exposure		Number of participants at risk	Loss to follow-up (n=56), n (%)	Number of outcomes without imputation (n=17), n (%)	Number of weighted outcomes after imputation (95% CI)	4-week uptake, participants (95% CI)	Pooled risk difference (95% CI)	Pooled risk ratio (95% CI)
**Overall**								
	CBT^a^ arm	469	19 (34)	7 (41)	7.433 (1.907 to 12.959)	0.016 (0.004 to 0.028)	0.003 (–0.013 to 0.018)	1.207 (0.406 to 3.590)
	Inoculation arm	466	20 (36)	4 (24)	4.200 (0.107 to 8.293)	0.009 (0.000 to 0.018)	–0.004 (–0.018 to 0.010)	0.687 (0.194 to 2.436)
	Standard arm	468	17 (30)	6 (35)	6.133 (1.239 to 11.027)	0.013 (0.003 to 0.024)	Reference	Reference
**Mental health symptoms―yes**								
	CBT arm	178	7 (12)	5 (29)	5.233 (0.626 to 9.841)	0.029 (0.004 to 0.055)	0.018 (–0.012 to 0.048)	2.583 (0.499 to 13.377)
	Inoculation arm	176	9 (16)	1 (6)	1.133 (–1.064 to 3.331)	0.006 (–0.006 to 0.019)	–0.005 (–0.025 to 0.015)	0.550 (0.050 to 6.048)
	Standard arm	179	5 (9)	2 (12)	2.033 (–0.782 to 4.849)	0.011 (–0.004 to 0.027)	Reference	Reference
**Mental health symptoms―no**								
	CBT arm	291	12 (21)	2 (12)	2.200 (–0.817 to 5.217)	0.008 (–0.003 to 0.018)	–0.007 (–0.024 to 0.011)	0.527 (0.097 to 2.850)
	Inoculation arm	290	11 (20)	3 (18)	3.067 (–0.399 to 6.532)	0.011 (–0.001 to 0.023)	–0.004 (–0.022 to 0.015)	0.745 (0.167 to 3.332)
	Standard arm	289	12 (21)	4 (24)	4.100 (0.092 to 8.108)	0.014 (0.000 to 0.028)	Reference	Reference

^a^CBT: cognitive behavioral therapy.

### Secondary Outcome: Vaccination Willingness at 4 Weeks

At 4 weeks, approximately one-third of respondents reported being very willing to receive another dose of the COVID-19 vaccine**―**30.7% (144/469) in the CBT kernel arm, 38% (177/466) in the inoculation arm, and 31%% (145/468) in the standard arm (Table 2). There were no significant differences in willingness across arms at the 4-week follow-up**―**participants in the CBT kernel arm were 0.3% less willing to get vaccinated than those in the standard arm (RD=−0.3%, 95% CI –6.3% to 5.8%; RR=99.2%, 95% CI 78.5%-125.3%), and participants in the inoculation arm were 7% more willing to get vaccinated than those in the standard arm (RD=7%, 95% CI 0.8%-13.3%; RR=122.7%, 95% CI 98.1%-153.4%). There were no differences in vaccination willingness at the 4-week follow-up when comparing those with and without anxiety or depression symptoms. The CBT kernel intervention group with mental health symptoms had an RD of 0.7% (95% CI −9.2% to 10.6%) and an RR of 102.1% (95% CI 71%-146.7%) compared to the standard arm. The inoculation group showed an RD of 7.4% (95% CI −2.8% to 17.7%) and an RR of 122.6% (95% CI 86.3%-174.1%) relative to the standard arm.

**Table 3:** Secondary outcome―vaccination willingness at the 4-week follow-up by intervention arm and presence of mental health symptoms.

Exposure		Number of participants at risk	Loss to follow-up (n=56), n (%)	Number of actual outcomes without imputation (n=445), n (%)	Number of weighted outcomes after imputation (95% CI)	4-week uptake, participants (95% CI)	Pooled risk difference (95% CI)	Pooled risk ratio (95% CI)
**Overall**								
	CBT^a^ arm	469	20 (36)	138 (31)	144.0 (121.4 to 166.5)	0.307 (0.259 to 0.355)	–0.003 (–0.063 to 0.058)	0.992 (0.785 to 1.253)
	Inoculation arm	466	21 (38)	168 (37.8)	177.0 (152.0 to 201.9)	0.380 (0.326 to 0.433)	0.070 (0.008 to 0.133)	1.227 (0.981 to 1.534)
	Standard arm	468	18 (32)	139 (31.2)	144.9 (122.2 to 167.6)	0.310 (0.261 to 0.358)	Reference	Reference
**Mental health symptoms―yes**								
	CBT arm	178	7 (12)	58 (13)	59.7 (45.4 to 74.1)	0.335 (0.255 to 0.416)	0.007 (–0.092 to 0.106)	1.021 (0.710 to 1.467)
	Inoculation arm	176	9 (16)	67 (15.1)	71.0 (55.3 to 86.6)	0.403 (0.314 to 0.492)	0.074 (–0.028 to 0.177)	1.226 (0.863 to 1.741)
	Standard arm	179	5 (9)	57 (12.8)	58.9 (44.5 to 73.2)	0.329 (0.249 to 0.409)	Reference	Reference
**Mental health symptoms―no**								
	CBT arm	291	13 (23)	80 (18)	84.2 (66.9 to 101.6)	0.289 (0.230 to 0.349)	–0.008 (–0.084 to 0.068)	0.972 (0.716 to 1.319)
	Inoculation arm	290	12 (21)	101 (22.7)	106.0 (86.7 to 125.3)	0.366 (0.299 to 0.432)	0.068 (–0.011 to 0.146)	1.227 (0.919 to 1.639)
	Standard arm	289	13 (23)	82 (18.4)	86.1 (68.5 to 103.7)	0.298 (0.237 to 0.359)	Reference	Reference

^a^CBT: cognitive behavioral therapy.

### Post Hoc Stratification Analyses of Primary and Secondary Outcomes

There were no significant differences by age, worry about COVID-19, or pretrial perceptions of vaccine efficacy for the primary outcome. However, individuals who were worried about COVID-19 and were in the inoculation arm had higher vaccination willingness compared to controls (RR=1.36, 95% CI 1.04-1.79; estimate available upon request). There were no significant differences by age or pretrial perceptions of vaccine efficacy for the secondary outcome.

### Sensitivity Analysis

The sensitivity analysis results (Tables S1-S3 in Multimedia Appendix 5) were largely consistent with the primary analysis (Tables 2 and 3), demonstrating similar vaccine uptake and vaccination willingness across intervention arms. The 4-week vaccine uptake rates remained stable, with minimal differences in RR and RD between the 2 analyses. For instance, the RR for vaccine uptake in the CBT versus standard arm and inoculation versus standard arm was 1.172 (95% CI 0.394-3.487) and 0.677 (95% CI 0.191-2.399), respectively, in the sensitivity analysis (Table S1 in Multimedia Appendix 5), closely aligning with 1.207 (95% CI 0.406-3.59) and 0.687 (95% CI 0.194-2.436) in the primary analysis, respectively. Similarly, vaccination willingness in the CBT versus standard arm and inoculation versus standard arm showed an RR of 0.996 (95% CI 0.787-1.26) and 1.224 (95% CI 0.978-1.532), respectively, in the sensitivity analysis compared to 0.992 (95% CI 0.785-1.253) and 1.227 (95% CI 0.981-1.534) in the primary analysis, respectively. Minor differences in CIs and complete case counts were observed, likely due to variations in handling missing data, but did not affect the conclusions. These findings supported the robustness of the primary analysis and confirmed that the intervention effects remained stable across different analytical approaches.

## Discussion

### Principal Findings

This randomized controlled trial (RCT) tested 2 novel COVID-19 vaccine messaging strategies—CBT kernels and inoculation theory—against standard public health messaging among individuals who had received at least one previous dose and were eligible for another. We found no significant differences in vaccine uptake or vaccination willingness across arms, including among participants with or without mental health symptoms. These findings underscore the difficulty of influencing vaccine uptake even among previously vaccinated individuals and those at higher risk of severe outcomes.

Inoculation theory–based messaging has rarely been evaluated in RCTs for vaccine promotion [4,5], although observational studies suggest that it may reduce receptivity to misinformation, a possible pathway to greater vaccination willingness [11]. Only 1 similar trial has tested this approach for vaccine uptake with inconclusive findings, underscoring the need for further research [4]. Given that our study took place years after the pandemic, the utility of inoculation messaging may be limited as the approach is designed to pre-empt misinformation before opinions are formed. While we identified a common narrative around vaccine effectiveness to guide messaging [17], entrenched beliefs may require more tailored strategies. Still, post hoc stratification analyses among those worried about COVID-19 showed that inoculation messaging may hold promise to increase vaccination willingness. Follow-up at 6 months will further explore its impact on vaccine uptake.

To our knowledge, this is the first study to develop and test vaccine uptake messaging using CBT kernels. Several studies have assessed the efficacy of CBT interventions on common mental health symptomatology during the pandemic [25] and advocated for simultaneous evidence-based mental health treatment with COVID-19 public health messaging [13]; however, the use of CBT kernels to encourage adherence to COVID-19 prevention measures among individuals with common mental health symptoms has not been explored. Although we did not identify differences in vaccine uptake according to anxiety or depression symptomatology even among those in the CBT arm, this approach to developing non–symptom-related public health messaging for individuals with common mental health symptoms is novel and should be explored further. Future research should refine this approach to public health messaging by using motivational interviewing strategies [26] during the pilot-testing phase to improve the messaging and effectiveness of such strategies.

Three large (N>100,000) RCTs grounded in *nudge theory* examined the effects of SMS text message reminders on vaccine uptake. Two found small but statistically significant increases in COVID-19 vaccination [4,27], whereas the other large trial did not [28]. Those trials that did find an effect observed 1% to 2% absolute increases in COVID-19 vaccine uptake from low levels at baseline. The framing of the messaging was examined in one of these trials, showing that messages framed around ownership (eg, *your vaccine is ready and waiting for you*) providing a specific date, time, and location are most effective. While encouraging, these trials did not identify strategies capable of large absolute increases in COVID-19 vaccination among adults in the United States.

### Study Limitations

Our study was conducted outside the traditional respiratory virus season, well after the latest vaccines became available and in a period of relatively low COVID-19 risk**―**during the fielding period, every jurisdiction in the country reported low levels of respiratory illness activity, and most key indicators of COVID-19 activity remained minimal [29,30]. This may have influenced the limited vaccine uptake among participants. Follow-up was carried out in November 2024, coinciding with the release of the 2024 vaccine formulations. The analysis of results is still ongoing and may provide insights into long-term effects of the intervention during higher-transmission periods. In addition, given that this study was conducted several years after the pandemic, the interaction of anxiety, depression, and COVID-19 vaccination may differ from that observed at earlier stages of the pandemic.

Furthermore, there are limitations to the interventions themselves. While video length was kept short and consistent across arms to maintain attention and align with typical media formats, this brevity may have constrained the ability to fully distinguish between communication approaches. Longer content might have enhanced the impact of each strategy. Participants may also have responded differently to the information presented had the actors in the videos been different. In addition, although participants were required to remain on the video page for its duration before proceeding, the study team could not confirm whether they actually watched the videos. Even though we had initially planned to conduct follow-up interviews with the 1.2% (17/1403) of the participants who got vaccinated, funding constraints at the time prevented us from carrying them out. This limited our ability to explore in greater depth which specific aspects of the intervention may have influenced vaccination decisions.

An important limitation of this study is the low overall vaccine uptake rate (17/1403, 1.2%) and the minimal absolute differences in uptake across study arms. Although the sample size calculation was based on historical data and powered to detect a 2.5–percentage point difference, the actual differences observed were smaller, likely rendering this study underpowered to detect such modest effects. This limitation should be considered when interpreting the null findings as this study may not have been adequately powered to identify small but potentially meaningful changes in behavior. In addition, the low baseline uptake likely reflects contextual factors at the time of the study (eg, perceived risk, vaccine availability, and public health messaging), which may limit the generalizability of these findings to periods with higher vaccine demand or different public health conditions. Moreover, although this study purposively sampled participants with anxiety or depression symptoms and a prespecified power analysis was conducted (Multimedia Appendix 4), the subgroup analysis was likely underpowered due to the overall low vaccine uptake observed. As a result, the lack of significant findings in this key subgroup should be interpreted with caution. Future studies with larger samples or higher outcome rates may be better positioned to detect meaningful intervention effects among individuals with anxiety and depression.

### Public Health Implications

This RCT tested 2 novel COVID-19 vaccine messaging strategies—CBT kernels and inoculation theory—against standard public health messaging among individuals who had received at least one COVID-19 vaccine. This study contributes to the literature by operationalizing and testing 2 theoretical messaging strategies, yet the results highlight the complexities of addressing vaccine hesitancy in a population with entrenched beliefs. Although inoculation theory–based messaging showed some promise in subgroup analysis, particularly among individuals still concerned about COVID-19, further investigation is needed to tailor these messages more effectively. Similarly, while the CBT kernels aimed to reduce psychological barriers to vaccination, the brief, stand-alone format may not be enough to shift behavior. The CBT methodology is not usually a one-time experience but requires ongoing engagement with a participant. More broadly, the findings highlight the difficulty of translating theory into practice, particularly when trying to translate complex behavioral frameworks such as CBT into short, scalable public health messages. More research is needed to understand the practical applications of this theory. Ultimately, the findings suggest that existing messaging strategies may be insufficient to drive substantial changes in vaccine uptake and emphasize the need for continued exploration of more targeted and personalized communication interventions.

In addition, the null findings underscore systemic challenges in the broader context of COVID-19 vaccination, including the transition from an acute pandemic to a more ambiguous, seasonal vaccination landscape. Messaging regarding the need for ongoing COVID-19 vaccines has been inconsistent, and many people remain unclear about why and when they need them, compounded by growing mistrust and shifting public perceptions. Future research should refine these approaches and assess their long-term effects on vaccination behavior, whereas testing during high-transmission periods may also enhance generalizability.

Due to a shift in US federal research priorities, the National Institutes of Health grant that supported this work was terminated and later reinstated as part of an ongoing lawsuit before the project could be completed. This has greatly constrained our ability to conduct additional planned analyses and follow-up work. Nonetheless, our findings underscore the urgent need for continued research on strategies to promote vaccine uptake in ways that can reduce severe disease and death, including reducing the impact of mis- and disinformation, with attention to those with mental health symptoms. Although our trial did not find meaningful differences in vaccine uptake between study arms at 4 weeks, testing these theory-informed approaches adds to the growing body of evidence on what may—and may not—be effective. Future research should continue to explore, refine, and rigorously evaluate communication strategies that address informational and behavioral barriers to vaccination, including long-term effects on vaccination behavior, especially in the face of persistent disinformation and declining trust in public health.

## Appendix

Multimedia Appendix 1 Intervention details.

Multimedia Appendix 2 Study measures.

Multimedia Appendix 3 Sensitivity analyses.

Multimedia Appendix 4 Imputation methods.

Multimedia Appendix 5 Supplementary Tables S1-S3.

Multimedia Appendix 6 CONSORT checklist.

## Abbreviations

CBT: cognitive behavioral therapy
CHASING: Communities, Households, and SARS-CoV-2 Epidemiology
CONSORT: Consolidated Standards of Reporting Trials
ITT: intention-to-treat
RCT: randomized controlled trial
RD: risk difference
RR: risk ratio
SMD: standardized mean difference

## Data Availability

The datasets generated or analyzed during this study are available from the corresponding author on reasonable request.
