# Supplementary material for: Testing Theory-Enhanced Messaging to Promote COVID-19 Vaccination Among Adults: Randomized Controlled Trial
Source: J Med Internet Res. 2025 Oct 7;27:e79228. doi: 10.2196/79228 (PMC12541261; doi:10.2196/79228)
Supplement: Multimedia Appendix 1 [file jmir_v27i1e79228_app1.docx]

**Appendix A***.*

The videos were professionally created with Long Story Short, a production company with experience in PSA creation at local, national, and global public health organizations. Our research team worked closely with the production team throughout the process, iterating on scripts, refining takes, selecting actors, and providing feedback to ensure clarity and consistency. The research team included experts in mental health disorders, COVID-19 surveillance, infection and transmission, vaccine hesitancy and uptake, public health emergency preparedness and response, and risk communication. A member of the research team was present on set during filming to oversee production and ensure fidelity to our intended messaging. By creating all videos within the same production process, we maintained control over factors such as tone, format, duration, and overall presentation, reducing the risk of unintended differences that could influence participants’ responses.

**Videos**

Setting: This conversation takes place in a generic setting, softly reminiscent of a therapist’s office or doctor’s waiting room, but without clear signals to a medical office or distinct location. Medical professional and actor are seated, as if they are having a casual, private appointment.

Wardrobe

Medical Professional: Formal / business casual clothes that do not signal any particular profession or expertise.

Patient: Casual, nondescript clothing, that is less formal than the medical professionals.

Scripts

#1: Inoculation [00:48 seconds]

| **Visual** | **Dialogue** |
| --- | --- |
| *[Doctor & Patient are seated, having a casual conversation. We see them both in the first shot to establish the scene.]* |  |
|  | *[as if finishing a conversation]*  Doctor: Did you get one of the latest vaccines for COVID-19? |
|  | Patient: *[awkwardly, like they know they should have gotten it but haven’t]*. Uhh… no. I just haven’t had the time. I’ve been reading online and it doesn’t really do anything anyways. |
|  | Doctor: *[realizing what’s going on] [approachable, but firm]* There are people who don’t want you to get the vaccine. They’re going to tell you that the vaccine doesn’t work. But that’s just misinformation. It can confuse you, or mislead you. |
|  | Patient: But… the vaccine won’t stop me from getting COVID, right? |
|  | Doctor: It won’t, but it will stop you from getting really sick. And if you do get COVID, people who are vaccinated are 10 times less likely to go to the hospital than people who aren’t [4]. |
|  | Patient: Would definitely love to avoid that... |
|  | Doctor: *[sincerely]* Then, I suggest getting one of the COVID vaccines. |
| *[shot goes out of focus, but we see the doctor and patient continue conversation silently, as the Voiceover speaks]* | *(Voiceover)*  Doctor: If you see something that says the vaccine doesn’t work, remember, don’t be mislead by misinformation. The vaccine is safe, and it will prevent you from getting a bad case of COVID. Make sure to get yours. |
| LOGOS:  CUNY SPH  UNC  ISPH |  |

#2: Cognitive-behavioral Therapy [00:41 seconds]

| **Visual** | **Dialogue** |
| --- | --- |
| *[Patient and Doctor sit across from each other in a clean, professional space.]* |  |
|  | *[quick cold open - attention grabbing line]*  Patient: I don’t have time to get the COVID-19 vaccine. |
|  | Doctor: Is it that you don’t have time… *[raising an eyebrow]* or are you worried about it? |
|  | Patient: *[sheepishly admitting]* I’ve been reading online that it doesn’t do anything anyways. |
|  | Doctor: There’s a lot of misinformation out there about the vaccine… It can be overwhelming, which makes it easy to listen to something that confirms how we already feel. |
|  | Patient: *[repeating so they understand]* Like I don’t want to get the vaccine, so I’m listening to the people who tell me I don’t have to? |
|  | Doctor: Right! But I can tell you that the COVID-19 vaccine is safe and it does work. It will stop you from getting a really bad case of COVID [4]. |
|  | Patient: *[determined]* I think I need to get the vaccine. |
| *[shot goes out of focus, but we see the doctor and patient continue conversation silently, as the Voiceover speaks]* | *(Voiceover)*  Doctor: The vaccine will stop you from getting a bad case of COVID. Make sure you take the time to get yours. |
| LOGOS:  CUNY SPH  UNC  ISPH |  |

#2: Standard Public Health Messaging [00:36 seconds]

| **Visual** | **Dialogue** |
| --- | --- |
| *[Doctor and patient are finishing a visit]* |  |
|  | *[quick cold open - attention grabbing line]*  Doctor: Did you get one of the latest vaccines for COVID-19? |
|  | Patient: *[sincerely, friendly]* I didn’t know there was one available. |
|  | Doctor: There is! And you’re eligible to get it. The vaccine is safe to get, and while it won’t stop you from getting COVID, it will stop you from getting really sick. And if you get COVID, people who are vaccinated are 10 times less likely to have to go to the hospital than people who aren’t. So: why not avoid a serious case and make the time to get yours [4]. |
| *[shot goes out of focus, but we see the doctor and patient continue conversation silently, as the Voiceover speaks]* |  |
|  | *(Voiceover)*  Doctor: Protect yourself from a bad case of COVID, and keep everyone around you safe too. Make sure you get the latest COVID-19 vaccine. |
| LOGOS:  CUNY SPH  UNC  ISPH |  |

*Text Message Content*

1. **CBT Arm**: The COVID-19 vaccine is safe and will prevent you from getting a bad case of COVID. It’s worth taking the time to get yours. For help finding a COVID-19 vaccine near you, visit https://www.vaccines.gov/
2. **Inoculation Arm:** Don’t be misled by misinformation. Remember, the COVID-19 vaccine is safe and will prevent you from getting a bad case of COVID. Make sure to get yours. For help finding a COVID-19 vaccine near you, visit https://www.vaccines.gov/
3. **Standard Public Health Messaging Arm**: Protect yourself from a bad case of COVID. Keep everyone around you safe too. Make sure you get the latest COVID-19 vaccine. For help finding a COVID-19 vaccine near you, visit https://www.vaccines.gov/
