# Supplementary material for: Testing Theory-Enhanced Messaging to Promote COVID-19 Vaccination Among Adults: Randomized Controlled Trial
Source: J Med Internet Res. 2025 Oct 7;27:e79228. doi: 10.2196/79228 (PMC12541261; doi:10.2196/79228)
Supplement: Multimedia Appendix 2 [file jmir_v27i1e79228_app2.docx]

**Appendix B**

*Study Measures*

We captured participants’ age and healthcare-related measures at enrollment in the trial. Healthcare-related measures included the primary source of health insurance, whether a participant had access to a primary care doctor, and experience of healthcare related discrimination.

At enrollment into the CHASING COVID cohort, we measured gender, sex, race and ethnicity, education, household income, and presence of children under 18 in the household. Susceptibility to severe COVID-19 given SARS-CoV-2 infection, employment status, state of residence, area-level political affinity, number of COVID-19 vaccinations, and number of prior SARS-CoV-2 infections were defined using the most current information from follow-up in the CHASING COVID cohort.

Participants were considered to have conditions that increase risk of severe COVID-19 if SARS-CoV-2 infected, if they were aged 60 or older at trial enrollment, or if they were smokers or had any chronic conditions, based on the most current information from the CHASING COVID Cohort. Chronic conditions included chronic lung disease, current asthma, type 2 diabetes, heart conditions, kidney disease, or immunocompromised conditions [17].

Concern about COVID-19 was defined as participants answering "somewhat worried" or "very worried" to either of the following questions at trial enrollment: "How worried are you about getting sick from COVID-19?" or "How worried are you about your loved ones getting sick from COVID-19?" Participants were considered to be misinformed about vaccine efficacy if they disagreed with any of the following statements at trial enrollment: "I need the COVID vaccine even if I’ve already had COVID," "The COVID vaccine will protect me from being hospitalized with COVID," or "Staying up to date on COVID vaccine shots is important to stay protected from severe COVID disease." Details on the distribution of participant characteristics by arm can be found in the following Table 4.
