# Supplementary material for: Testing Theory-Enhanced Messaging to Promote COVID-19 Vaccination Among Adults: Randomized Controlled Trial
Source: J Med Internet Res. 2025 Oct 7;27:e79228. doi: 10.2196/79228 (PMC12541261; doi:10.2196/79228)
Supplement: Multimedia Appendix 3 [file jmir_v27i1e79228_app3.docx]

**Appendix C**

A series of sensitivity analyses were conducted to address assumptions related to loss to follow-up and protocol adherence, including complete case analysis, inverse probability weighting, per-protocol analysis for participants fully adhering to the intervention, and multiple imputation for missing outcomes, with consistent results observed across primary and secondary outcomes.

*Sensitivity Analyses*

1. As a sensitivity analysis of the loss to follow-up assumptions in the multiple imputation model, we conducted the following sensitivity analysis.1) We omitted participants without outcome data and conducted a complete case analysis. This approach is valid if outcome data is missing completely at random (Supplemental Table 1). 2) We used estimating-equation methods to weight complete cases (i.e., participants with outcome data) by the inverse of an estimate of the probability of being observed (Supplemental Table 2) [33]. The probability of an outcome being observed was modeled using pre-randomization and post-randomization factors predictive of outcomes and dropout: study arm, pre-randomization mental health status, age category, residential geographical region, access to a personal provider, discrimination experience, long COVID status, most recent vaccination timing, flu vaccine receipt. We note that the two sensitivity analyses of missing outcome data aren’t *strictly* ITT analyses as randomized participants are excluded.
2. As a sensitivity analysis of the assumption of perfect implementation and adherence to the protocol, we analyzed data under the per-protocol principle (Supplemental Table 3). We restricted to all participants who were fully adherent to intervention implementation: reported the video held their attention. As exclusion of participants from the analysis compromises randomization, the per-protocol analysis is similar to a non-randomized observational design; therefore, per-protocol analysis requires adjustment for confounding due to incomplete adherence to randomization (treatment), and adjustment for any selection bias due to loss to follow-up [25,33]. For the primary outcome, we generated risk ratios using a robust Poisson regression model. The Poisson model adjusted for a priori factors assumed to confound adherence to full implementation and the vaccination at 4-weeks. This included age, having children under 18 in the household, education, vaccine hesitancy, GAD-7 scores, PHQ-8 scores, and the post-randomization factor includes the day of the week. To address missing outcome data, we used multiple imputation as described above.

*Sensitivity Analysis of Primary and Secondary Outcomes*

The analysis results were similar across all sensitivity analyses: 1) restricting to the 1,357 participants who completed the four-week survey, 2) using an inverse probability of censor weighted (IPCW)-adjusted model, 3) restricting to the N = 1,304 participants who reported that they watched the intervention video.
