# Supplementary material for: Testing Theory-Enhanced Messaging to Promote COVID-19 Vaccination Among Adults: Randomized Controlled Trial
Source: J Med Internet Res. 2025 Oct 7;27:e79228. doi: 10.2196/79228 (PMC12541261; doi:10.2196/79228)
Supplement: Multimedia Appendix 4 [file jmir_v27i1e79228_app4.docx]

**Appendix D**

*Imputation Methods*

To conduct multiple imputation of missing outcome data, we assumed that participants lost to 4-week follow-up were missing at random. We chose to align the imputation model with assumptions consistent with the design of a randomized controlled trial. Specifically, we included only the randomized trial arms and the stratification factor (with and without symptoms of anxiety and depression) as predictors in the imputation model, in order to preserve the integrity of the randomization structure. We conducted 30 imputations and combined the parameter estimates i.e., regression coefficients, from each imputed dataset by averaging. We combined the variances using Rubin's rules [37,38] which account for both within-imputation variance and between-imputation variance. By pooling the results, we obtained overall estimates and standard errors that reflect the uncertainty due to missing data.

*Sample Size Calculation*

The initial power calculations were based on an expected sample of 1,500 (n=1,000 experimental arms vs 500 control arm) and historical data on COVID-19 vaccine uptake. Over a four-week period in the Spring of 2022, the percentage of people who received at least one dose of the COVID-19 vaccine increased by 1.3% [20]. With alpha =0.05 and a sample of 1,500, we predicted having at least 80% power to detect an absolute difference in vaccine uptake as small as 2.5 percentage points (e.g., 3.8 vs 1.3, corresponding relative risk [RR] = 2.9). We also estimated that we would have 305 participants with moderate to severe symptoms of anxiety or depression in each arm (61% of all trial-enrolled participants). We predicted that we would have 80% power to detect a difference as small as 5.3 percentage points in vaccine uptake, assuming alpha = 0.05 and a two-sided test. Power calculations were updated based on the final enrollment of N = 1,403 participants. The revised detectable effect size was 3.07).
